# Coincidence of Concentric Vessel-Wall Contrast Enhancement in Moyamoya Disease and Acute Postoperative Ischemic Stroke During Revascularization Procedures

**DOI:** 10.3390/brainsci14121190

**Published:** 2024-11-26

**Authors:** Patrick Haas, Till-Karsten Hauser, Lucas Moritz Wiggenhauser, Leonie Zerweck, Marcos Tatagiba, Nadia Khan, Constantin Roder

**Affiliations:** 1 Department of Neurosurgery and Moyamoya Center, University of Tübingen, Hoppe-Seyler-Straße 3, 72076 Tübingen, Germany; lucas.moritz.wiggenhauser@med.uni-tuebingen.de (L.M.W.); marcos.tatagiba@med.uni-tuebingen.de (M.T.); nadia.khan@kispi.uzh.ch (N.K.); constantin.roder@med.uni-tuebingen.de (C.R.); 2 Department of Neuroradiology, University of Tübingen, Hoppe-Seyler-Straße 3, 72076 Tübingen, Germany; till-karsten.hauser@med.uni-tuebingen.de (T.-K.H.); leonie.zerweck@med.uni-tuebingen.de (L.Z.); 3 Moyamoya Center, University Children’s Hospital Zurich, University of Zurich, Steinwiesstrasse 75, 8032 Zurich, Switzerland

**Keywords:** cerebral revascularization, Moyamoya disease, postoperative ischemic stroke, contrast-enhanced vessel wall imaging

## Abstract

Background: Concentric vessel-wall contrast enhancement (VW-CE) of the terminal carotid artery and its proximal branches may be linked to ischemic strokes, disease activity and progression in Moyamoya disease (MMD). The objective of this retrospective cohort study is to analyze the association between VW-CE and perioperative acute ischemic stroke (PAIS) occurring within 24 h after revascularization. Methods: All previously untreated MMD patients who required revascularization and who had undergone preoperative MRI with VW-CE-sequences were included. PAIS was detected by CT and/or diffusion-weighted MRI sequences within 24 h postoperatively. Results: Of the 110 patients included (female-to-male ratio: 2.7:1, median age: 45.1 (16.6–69.2); *n* = 247 revascularizations), a priori VW-CE was present in 67.3% (mean time from MRI to first surgery: 86 days ± 82 days). PAIS occurred in five patients undergoing primary revascularization (PAIS rate per revascularization: 2.1%), all of whom had a preoperative pathological VW-CE in the vascular segment corresponding to the stroke area. Two (40%) incidents of PAIS occurred in revascularized territory, while three (60%) occurred in non-revascularized vascular territory. In each case, the supplying artery exhibited VW-CE, indicating disease activity. No additional PAIS occurred during subsequent revascularizations in cases of multistage procedures (*n* = 38), such as ACA or PCA revascularization as a second step. Conclusions: Preoperative VW-CE in one or more vascular segments may be a marker for postoperative stroke in the respective vascular territory at the time of revascularization. VW-CE imaging should be routinely performed when planning revascularization in MMD. If VW-CE is found, strict perioperative monitoring of these high-risk patients should be performed to achieve the best results possible.

## 1. Introduction

Moyamoya disease (MMD) can cause ischemic stroke due to alterations in the vascular architecture of the terminal segments of the internal carotid arteries (ICAs), proximal anterior cerebral artery (ACA) or middle cerebral artery (MCA), as perfusion deficits can no longer be compensated for by an insufficient rete mirabile. If indicated, revascularization with extra-/intracranial (EC-IC) bypasses is regarded as the gold standard of therapy, as it has been demonstrated to reduce the recurrence rate of stroke in hemodynamically insufficient patients [1]. However, ischemic strokes may also occur perioperatively, although the precise pathomechanism is not yet fully understood. Several studies have investigated the risk and protective factors for perioperative acute ischemic stroke (PAIS) after revascularization procedures in patients with MMD, mainly focusing on comorbidities [2,3,4,5,6,7]. The potential pathomechanisms underlying this phenomenon have been subject to considerable debate: among those discussed are hemodynamic effects due to a vascular steal or watershed shift after direct revascularization on the one hand, as well as altered cerebral blood flow caused by anesthetics and changes of arterial blood pressure on the other [8,9,10]. A potential correlation between the vascular territory affected by PAIS and MMD remains uncertain, as postoperative strokes may also occur in the hemisphere contralateral to the operated side [11,12]. As PAIS is potentially one of the most serious complications in the surgical treatment of MMD, a deeper understanding is crucial for improving patient outcomes.

Vessel-wall imaging in MMD has recently received increased scientific attention [13,14]. Contrast enhancement in the affected vessel walls (VW-CE) may be an indicator of disease activity in the vessel wall [15]. Vessel-wall alterations in MMD have been previously identified through autopsy studies, and they are characterized by proliferating smooth muscle cells and macrophage infiltration [16]. Although the significance of VW-CE is not yet fully understood, studies have indicated that contrast uptake in affected vessel walls may predict disease progression and increase the risk of poor patient outcomes [15,17,18,19]. Consequently, MRI screening for VW-CE is routinely performed in all MMD patients at our center. This study retrospectively investigates the occurrence of PAIS during EC-IC revascularization in MMD and its correlation with preoperative VW-CE imaging findings.

## 2. Methods

### 2.1. Inclusion and Exclusion Criteria

MMD was defined according to the Japanese Guidelines for the Management of Moyamoya disease [20]. Diagnosis and therapy were based on the consensus recommendations of the European Stroke Organization [21]. The study included adult MMD patients who had not previously undergone revascularization and who were treated with EC-IC bypass for the first time at our center between 12/2013 and 12/2023. Only patients who had received preoperative high-resolution MRI with contrast-enhanced fat-saturated vessel-wall imaging were included in this study. Patient files were scanned for clinical signs of ischemic stroke and imaging was used to confirm PAIS (MRI and/or CT) with an onset < 24 h after surgery. In general, all patients underwent routine postoperative imaging by CT or MRI within 24 h after revascularization.

### 2.2. VW-CE MR Imaging Protocol

VW-CE MRI was performed on a 3Tesla scanner (Magnetom Vida Fit, Siemens Healthineers, Forchheim, Germany) with a 64 Channel Head/Neck Coil. The sequence parameters were: 3D T1 SPACE (Sampling Perfection with Application optimized Contrasts using different flip-angle evolution) with a time of repetition (TR) of 600 ms, time of echo (TE) of 23 ms, flip angle of 120°, slice thickness of 0.8, field of view (FOV) of 185 × 220 mm, a time of acquisition (TA) of 3:45 and a 384 × 324 matrix, resulting in a voxel size of 0.29 × 0.29 × 0.80 mm. A contrast agent (dosage: 0.1 mmol Gadobutrol/kg) was administered during the measurement of a perfusion sequence (TA 2:29). The VW-CE sequence was started after a whole-brain T1 sequence (TA 1:46); so, the VW-CE imaging was started at around 4:25 min after the contrast administration. Blood suppression was achieved by inversion recovery, and no pulse gating was used.

### 2.3. Data Processing, Workflow and Statistics

All the data were stored in a custom database (REDCap) and statistically analyzed (JMP, SAS Institute, Cary, NC, USA). Levene’s test for homogeneity of variance was applied and pooled/unpooled t-tests were performed accordingly. *p*-values < 0.05 were considered significant. Contingency tables were analyzed with Fisher’s exact test. Nominal logistic regression models were used for multivariate analysis. A stepwise regression approach was used to select all the effects. Odds ratios (ORs) with 95% confidence intervals were calculated as a measure of the strength of association.

### 2.4. Severity of PAIS

The Clavien–Dindo classification (CDC) was used to assess the severity of the PAIS, which is standard for all complications in our clinic [22]. The modified Rankin Scale (mRS) and the National Institute of Health Stroke Scale (NIHSS) were recorded preoperatively and postoperatively to assess the clinical condition of the patient.

### 2.5. Standardized Diagnostic Protocol

As part of a standardized diagnostic protocol, patients suspected of having MMD undergo conventional angiography and CO_2_-evoked blood-oxygenation-level dependent (BOLD) MRI to determine vasoreactivity as a correlate of cerebral perfusion reserve, as previously described [23,24,25,26,27,28]. In cases of borderline findings, PET imaging with acetazolamide challenge is also performed. The indication for EC-IC revascularization is then determined on the basis of all the findings. For multi-territorial findings, revascularization of bilaterally affected MCA territories is performed in a single-stage procedure. Any additional affected ACA or PCA territories are then revascularized in secondary or tertiary procedures as part of a multi-stage tailored procedure, as described previously [25]. In the event of an initial ischemic stroke, a period of 6 to 12 weeks is typically allowed to elapse between its occurrence and the first revascularization in order to permit stabilization of the patient’s blood flow and clinical condition. This period of time is in accordance with the European consensus recommendations [21]. All patients are hydrated by intravenous administration of balanced full-electrolyte solutions for a minimum duration of 12 h prior to surgery. Antihypertensive medication, with the exception of beta-blockers, are discontinued during anesthesia. Perioperative blood pressure monitoring begins with an invasive arterial blood pressure measurement while the patient is still conscious to avoid blood pressure fluctuations due to the induction of anesthesia.

### 2.6. Standard Protocol Approvals, Registrations, and Patient Consent

All procedures performed in studies involving human participants were in accordance with the ethical standards of the institutional research committee and with the 1964 Helsinki Declaration and its later amendments or comparable ethics standards. Approval from the local ethics committee was obtained (105/2024BO2). Due to the retrospective character of this analysis, no specific formal consent from the participating patients was obtained.

## 3. Results

**Figure 1:**
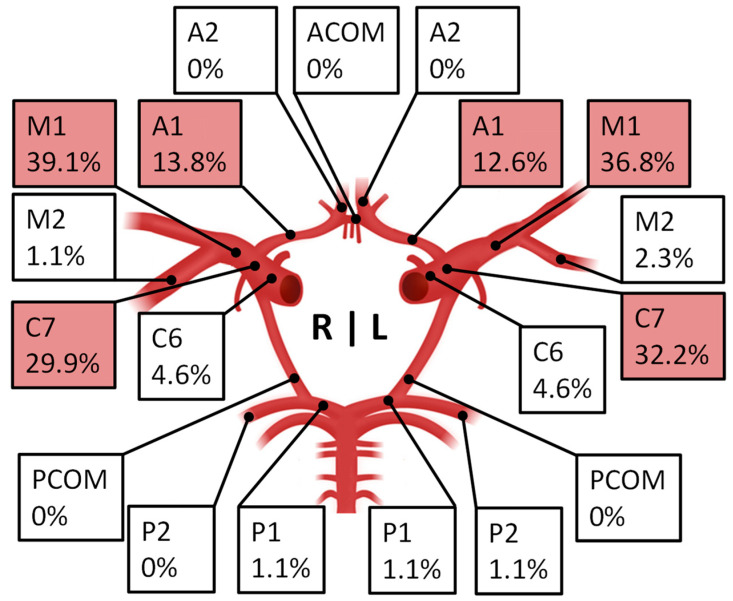
A total of 67.3% of all MMD patients with VW-CE imaging showed contrast enhancement of the vessel wall (VW-CE). The percentage prevalence of these VW-CEs in the different vessel segments of the arterial circle of Willis is shown (right hemisphere (R); left hemisphere (L)). (ICA: C6 and C7; MCA: M1 and M2; ACA: A1, ACOM and A2; PCA: P1, PCOM and P2). Note: Very rarely, VW-CE were also seen in the C5 section of the ICA; these are not shown for reasons of clarity.

Inclusion criteria were met by *n* = 110 patients. The female-to-male sex ratio was 2.7:1, and the median age was 45.1 years (16.6–69.2) (Table 1). A total of *n* = 247 vascular territories were revascularized, of which *n* = 157 were direct or combined (direct and indirect) bypasses (63.6%). The majority of patients underwent single-stage revascularization surgery (65.5%; unilateral or bilateral revascularization), while 31.8% of patients underwent two-stage revascularization and three patients underwent up to four stages of revascularization (all of them with secondary disease progression in a not-yet revascularized territory). The most common target regions for the first revascularizing procedure were the MCA territory on the right, with a total of *n* = 84, and on the left, with *n* = 82, followed by the ACA territory (right *n* = 36, left = 32). The mean time between preoperative VW-CE MRI and first revascularization procedure was 86 ± 82 d. The overall prevalence of contrast uptake in the arterial wall (VW-CE) was 67.3%. The distal C7 segment of the ICA (right 29.9%; left 32.2%) and the proximal branches A1 (right 13.8%; left 12.6%) and M1 (right 39.1%; left 36.8%) of the ACA and MCA were most frequently affected by VW-CE (Figure 1).

Unless otherwise specified, the value indicates the number of patients and the corresponding percentage.

### Perioperative Acute Ischemic Stroke After Revascularization

**Figure 2:**
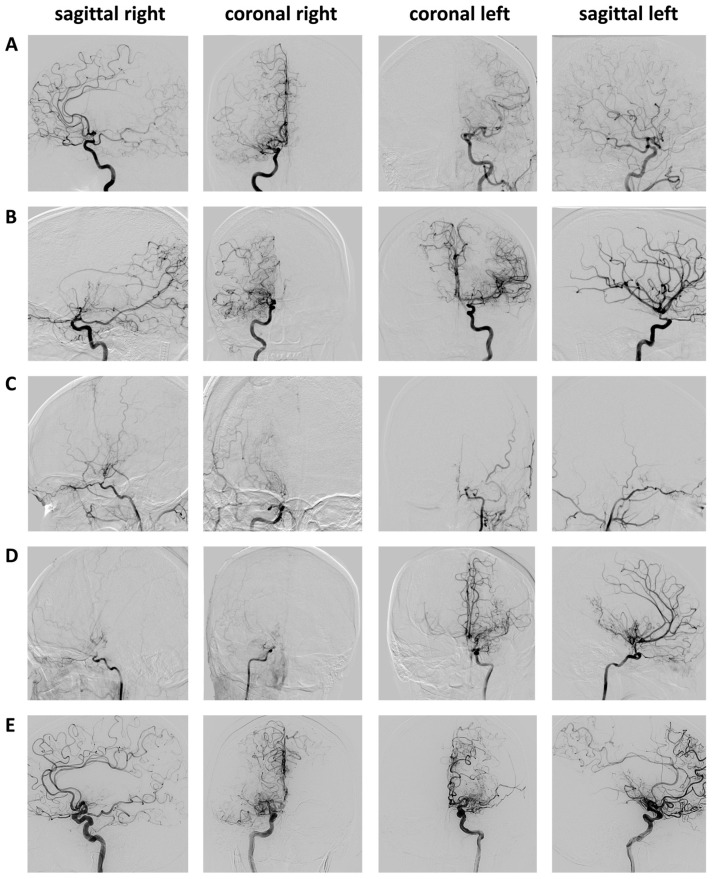
Preoperative angiograms of the five patients (**A**–**E**) with PAIS. (**A**) ACA supply on both sides via the right ICA. (**B**) Bilateral ACA supply via the left ICA. See the corresponding infarct pattern in Figure 3B. (**C**) Advanced ICA stenoses on both sides. (**D**) Right ICA occlusion, left hemispheric A1 stenosis and M1 occlusion. A matching PAIS pattern was also seen bilaterally in the ACA territory, with VW-CE of the left ICA. Secondary findings: two supraophthalmic ICA aneurysms on the left side. (**E**) Left ICA with collateralization to parietooccipital, consistent with PAIS (Figure 3E). Note: Angiograms of the basilar artery are not shown for reasons of clarity.

**Figure 3:**
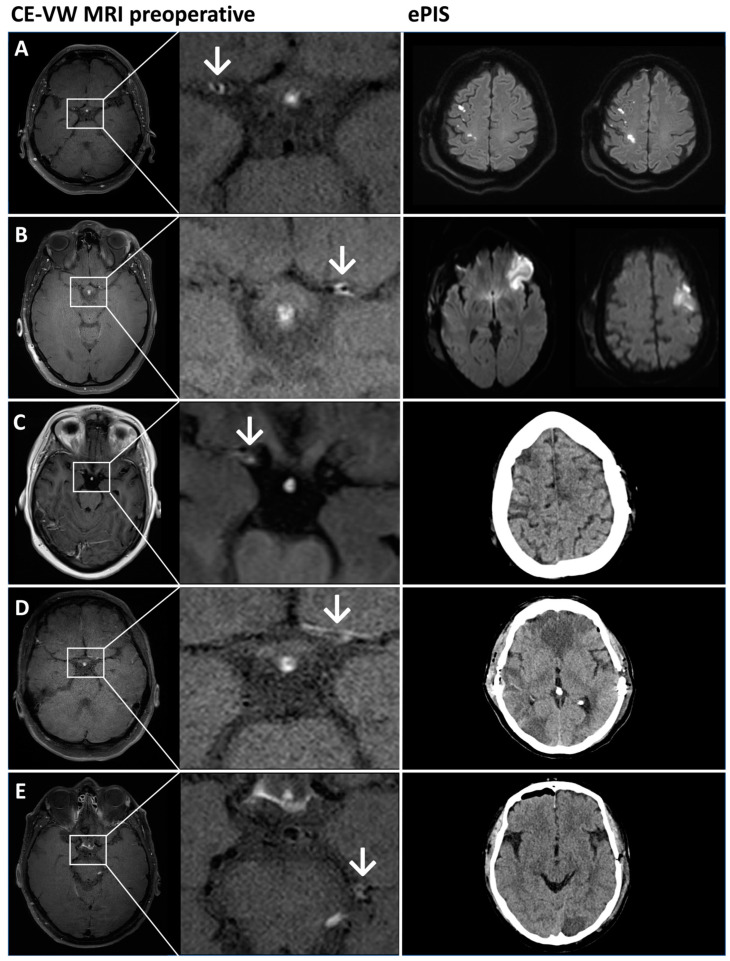
Imaging of five patients (**A**–**E**) who had a PAIS. Left column: preoperative VW-CE imaging (MRI). White arrows mark a VW-CE (exemplary slices). Right column: postoperative imaging to visualize a PAIS. The first two cases (**A**,**B**) received diffusion-weighted MRI sequences, while the other (**C**–**E**) received CT imaging. (**A**) Ischemia in the MCA and ACA/MCA watershed area with bilateral ACA supply via the right ICA (Figure 2A), which is possibly hemodynamically induced. (**B**) The initial situation is analogous to that of case (**A**), with bilateral ACA supply via the left ICA. However, in this instance, the infarcts are wedge-shaped, which may be indicative of a thromboembolic process. (**C**) Right frontal ischemia in preoperatively advanced ICA stenosis on both sides. (**D**) Territorial infarcts of the ACA on both sides and parietooccipital with ACA supply on both sides via the left ICA, corresponding to VW-CE, which is most likely hemodynamically caused by intraoperative blood pressure fluctuations. (**E**) Occipital ischemia on the left and collaterals (Figure 2E) via the left ICA with VW-CE at the P1/P2 transition.

The overall incidence rate of PAIS (*n* = 5) per bypass (*n* = 247) was 2.1%, 3.3% per revascularizing operation (*n* = 152) and 4.5% at the first surgery (*n* = 110). When only looking at patients with VW-CE, the PAIS rate at the first surgery was 6.8%. Figure 2 depicts the preoperative angiograms of patients with PAIS. All five cases of PAIS occurred in vascular territory supplied by a vessel affected by VW-CE: PAIS in the ACA or MCA territory (*n* = 4) occurred in the ipsilateral C7, A1 or M1 segment affected by CE-VW. One PAIS occurred in the PCA territory, which corresponded to a VW-CE in the ipsilateral P1 and P2 segments (Figure 3). Only three of the five cases of PAIS (60%) occurred in the revascularized territory itself. The severity of PAIS according to the Clavien–Dindo Classification (CDC) was ≤grade 2 in *n* = 4 cases and grade 5 in *n* = 1 case. This case showed a poor outcome due to bilateral infarction in the ACA territory (Figure 3B). The remaining cases exhibited only a modest decline in the NIHSS and mRS, accompanied by stable, or even improved, progression at the 6-month follow-up (Table 2). After univariate analysis of the comorbidities of smoking, diabetes mellitus (DM), alcohol consumption, body mass index (BMI) and patient characteristics (age, sex, ethnicity, initial disease manifestation asymptomatic vs. TIA vs. ischemic vs. hemorrhagic stroke), only increased BMI showed a significant association with PAIS (BMI (non-PAIS) 27.2 ± 5.4 vs. BMI (PAIS) 32.8 ± 6.5; *p* = 0.0273; OR = 1.2 95%CI 1.0–1.4). In a multivariate analysis of these variables, again, only BMI could be identified as a risk factor for PAIS. The occurrence of VW-CE was not significantly associated with PAIS.

## 4. Discussion

This study is the first to investigate perioperative acute ischemic stroke (PAIS) in MMD patients following revascularization, employing a novel imaging perspective. This perspective is based on the clinical observation of a potential correlation between the contrast enhancement of vessels proximal to the stroke area upon preoperative baseline MRI. All patients with PAIS exhibited preoperative concentric contrast enhancement of the arterial walls of the terminal segments of the ICA and/or proximal branches of the ACA, MCA, or PCA supplying the territory affected by PAIS. This anatomical association has not previously been observed and requires further investigation. The occurrence of VW-CE was not significantly associated with PAIS in the studied cohort, which may be attributed to the generally low incidence of PAIS itself. Postoperative acute stroke rates vary in the literature, partly due to differences in the time intervals studied or because calculations were made per bypass rather than per operation in the case of multi-territorial revascularization in single-stage procedures. While some authors report PAIS within 24 h after surgery, as we do, others have identified PAIS with a latency of up to 7 days [5,29,30]. Our PAIS rate of 3.3% per revascularizing surgery and 2.1% per bypass seems plausible given the rates described in the literature, which range from 2.1% to 6.9% [2,3,5,29,31].

Although PAISs were generally quite rare in the relatively large sample analyzed, which limits stochastic analysis, this observation merits further attention. It is possible that a pathological VW-CE may correspond to a disease activity that may result in the deterioration and instability of perfusion distal to the affected vessel segment. A correlation between disease progression in MMD and VW-CE has already been previously demonstrated [15,19]. This finding is consistent with other studies that have demonstrated a correlation between postoperative stroke and the imaging progression of steno-occlusive changes or clinically unstable disease [2,19,32]. Several studies have provided support for this hypothesis, identifying preoperative ischemic events as a risk factor for PAIS [3,5,6,33,34]. All identified patients with PAIS initially presented with symptoms of an ischemic stroke. However, this was not demonstrated to be a significant risk factor for PAIS. It is noteworthy that no spatial correlation with the occurrence of PAIS has yet been established, as several authors have correctly described the occurrence of ischemic stroke in locations contralateral to the operated hemisphere or clearly distant from the surgical site [3,7,11,12]. Therefore, not all occurrences of PAIS can be explained by a watershed shift phenomenon, which could be seen as a paradoxical decrease in cerebral blood flow in the adjacent cortex near the site of local hyperperfusion, or a steal phenomenon with increased retrograde flow versus decreased antegrade flow [8,9]. The data presented here suggest such a spatial correlation between the VW-CE and the area affected by PAIS. This may be a potential factor influencing the sequence of multi-stage procedures and the urgency of planning patient-specific revascularization procedures. The time interval between VW-CE imaging and surgical treatment in this study should not diminish the significance of the results given the presumed long-lasting and durable nature of the VW-CE in MMD, which approximates to at least 24 months [17,19]. During this period, a slow intensity increase of VW-CE can be observed for approximately 12 months, followed by a decrease in intensity for another 12 months.

In our cohort, no further PAISs were observed in multi-stage procedures. This may be attributed to the protective effect of enhanced blood flow resulting from the initial revascularization or a stabilization of hemodynamics in segments with disease progression, as a decreasing rate of PAIS in secondary interventions has also been reported in other studies [3]. However, this may also be biased by the fact that the hemisphere or vascular territory with an assignable VW-CE is often the hemisphere with the most pronounced symptoms and a limited perfusion reserve, and it is therefore treated first in the primary intervention. In view of our study results, the question arises as to whether revascularization should be postponed due to an increased PAIS rate until the VW-CE has subsided. Studies specifically addressing this issue are not known. However, based on the current state of the evidence, the risk–benefit ratio should be clearly in favor of prompt revascularization as VW-CE indicates increased disease activity and, consequently, an elevated risk of stroke [15,17,19].

With regard to comorbidities, this study identified obesity as a risk factor for PAIS, while other studies have also identified diabetes mellitus as an independent vascular risk factor [3,4,35]. These findings are conclusive in that both conditions may be interdependent and have been shown to have an overall negative impact on the cerebral vasoarchitecture through microvascular dysfunction [36]. In addition, patients diagnosed with PAIS exhibited a higher incidence of significantly reduced high-density lipoprotein (HDL) levels [37]. There was no evidence in this study that ethnicity had an impact on VW-CE or PAIS. However, the low proportion of non-Caucasian patients in the cohort analyzed is a limiting factor. Whether the observations from this study are also present in Asian patients with MMD would certainly be an interesting question for future studies.

The findings of this study indicate a possible direct relationship between VW-CE and PAIS. Nevertheless, it is not feasible to ascertain a causal relationship and the origin of these ischemic strokes. It seems reasonable to suggest that a VW-CE vessel segment may act as an indicator of an actively progressing stenosis, with hemodynamic restriction occurring distal to it. This is corroborated by the increased susceptibility of MMD patients to perioperative fluctuations in blood pressure levels. Conversely, it is also possible that VW-CE segments could be the origin of thromboembolic infarcts, which has already been demonstrated to be a factor in MMD [38,39]. This is also supported by the effect of antiplatelet therapy (APT), as a reduction in the risk of PAIS with APT was found in at least some, but not all, studies [1,40,41]. Overall, APT is most likely to result in improved overall survival in MMD, while the risk of hemorrhage appears to be reduced [1,42]. However, the present study cannot make a conclusion about the effect of APT on PAIS, as all patients included received APT as standard. Thus, MMD may be a mixed picture of hemodynamic and thromboembolic ischemia.

This study did not specifically address intraoperative risk factors. However, as previously stated, the distinctive features of surgical procedures may be a pivotal factor in the development of PAIS in a territory at risk due to VW-CE. Hemodynamic variations due to anesthetic management with vasoreactive drugs and a hematocrit shift due to volume management have been identified as risk factors for PAIS [10]. Therefore, patient-specific blood pressure management both intraoperatively and postoperatively may reduce the risk of PAIS [31]. In addition, the carbon dioxide sensitivity of the cerebral vasculature must be taken into account, as an increased incidence of PAIS has been described, at least in ventilation-associated hypocapnia and hypercapnia [5,43]. This emphasizes the necessity for close coordination between the surgeon and anesthesiologist regarding, e.g., perioperative blood pressure levels and ventilation parameters.

### Limitations

This is a retrospective analysis, which always has the risk of a certain bias. Due to the rarity of perioperative stroke, the cohort examined is rather small in comparison to the entire cohort treated. Nevertheless, the risk of an underpowered analysis seems low given the large study population in total. Still, further investigations of larger populations should certainly be considered. We therefore expect to see higher data densities in the future as we continue our imaging protocol and aim to analyze the pathophysiological impact of vessel-wall contrast enhancement in a translational clinical trial. Moreover, the analysis of individual dynamics of contrast enhancement before surgery was not possible, as only one preoperative VW-CE imaging was performed for each of the patients. Furthermore, this study concentrated on the diagnosis of VW-CE and PAIS. However, it is not possible to draw conclusions regarding the causal relationships between these factors alone, and further research is required to ascertain the significance of VW-CE in MMD.

## 5. Conclusions

Acute perioperative ischemic stroke represents a rare but serious adverse event of revascularization procedures in MMD. The findings of our study indicate a potential correlation between PAIS and the preoperative observation of concentric contrast enhancement of the affected vessel walls. In several studies this vessel-wall contrast enhancement was found to be associated with disease progression in the respective vessel segment. Patients with VW-CE may be at an elevated risk for acute perioperative ischemic strokes and should be monitored meticulously. Further research is required to gain a deeper insight into the relationship between VW-CE and the progression of MMD. This could potentially lead to improved patient outcomes by determining the appropriate treatment, including the timing of surgery and the sequence of territories to be revascularized in cases of multi-territorial involvement. Additionally, a more comprehensive understanding of the underlying pathophysiological processes leading to Moyamoya disease could be achieved.

## Figures and Tables

**Table 1:** Basic patient characteristics.

Patient Characteristics	n (%)
Sex - female - male	80 (72.7%) 30 (27.2%)
Age (at first surgery)	median 45.1 y (16.6–69.2)
Ethnicity - Caucasian - Asian - Arabic	99 (90.0%) 10 (9.1%) 1 (0.9%)
Initial MMD onset - hemorrhagic stroke - ischemic stroke or TIA/PRIND - minor (e.g., headache) or incidental	7 (6.4%) 77 (70.0%) 26 (23.6%)
Moyamoya disease - unilateral left - unilateral right - bilateral	21 (19.1%) 16 (14.5%) 73 (66.4%)
Suzuki classification (Right/Left) - grade 1 - grade 2 - grade 3 - grade 4 - grade 5 - grade 6	5/4 11/9 27/34 22/22 16/16 8/9
vessel-wall imaging - VW-CE	110 (100%) 74 (67.3%)
Time interval between VW-CE imaging and the first surgery	mean 86 d ± 82 d

**Table 2:** PAIS patient characteristics.

Case	Age [Years]	Sex	Suzuki (Left/Right)	VW-CE	Revascularization	Ethnicity	BMI	Smoking	HTN	DM	Initial MMD Onset	NIHSS Preop/Postop/6mFU	mRS Preop/Postop/6mFU
A	63	female	3/3	C7 right A1 right	STA-MCA right combined	Caucasian	26.6	no	yes	no	ischemic	0/5/3	2/3/3
B	46	female	2/4	C7 left	STA-MCA right combined	Caucasian	37.3	no	yes	yes	ischemic	0/3/3	1/1/1
C	22	female	5/3	C7 right M1 right C7 left	STA-MCA bilaterally combined	Caucasian	41.9	no	yes	no	ischemic	3/4/3	2/3/2
D	46	female	4/6	C7 left M1 left A1 left	STA-MCA bilaterally direct	Caucasian	29.1	no	no	no	ischemic	2/32/ #	3/5/ #
E	59	male	4/4	P1 left P2 left	STA-MCA bilaterally direct	Caucasian	29.1	yes	yes	no	ischemic	0/4/ *	1/2 *

arterial hypertension (HTN), diabetes mellitus (DM), National Institute of Health Stroke Scale (NIHSS), modified Rankin Scale (mRS). Follow-up conducted 6 months after revascularization (6mFU); #—lost to follow-up; *—6mFU still pending.

## Data Availability

The original contributions presented in the study are included in the article, further inquiries can be directed to the corresponding author.

## Abbreviations

ACA	anterior cerebral artery
APT	antiplatelet therapy
BMI	body mass index
BOLD	blood-oxygenation-level dependent
CDC	Clavien–Dindo Classification
CT	computed tomography scan
DM	diabetes mellitus
EC-IC	extracranial–intracranial
PAIS	perioperative acute ischemic stroke
HDL	high-density lipoprotein
HTN	(arterial) hypertension
ICA	internal carotid artery
MCA	middle cerebral artery
MMD	Moyamoya disease
MRI	magnetic resonance imaging
OR	odds ratio
VW-CE	contrast-enhancing vessel wall
TIA	transitory ischemic attack
